# I-V Curves of an Apigenin Dye and Their Analysis by a New Parabolic Function

**DOI:** 10.3389/fchem.2021.643578

**Published:** 2021-08-05

**Authors:** Kayode Sanusi, Olukayode S. Ajayi, Adegoke O. Borisade, Regina B. Elusiyan, Yusuf Yilmaz, Ümit Ceylan

**Affiliations:** ^1^ Department of Chemistry, Obafemi Awolowo University, Ile-Ife, Nigeria; ^2^ Centre for Energy, Research and Development, Obafemi Awolowo University, Ile-Ife, Nigeria; ^3^ NT Vocational School, Gaziantep University, Gaziantep, Turkey; ^4^ Department of Medical Services and Techniques, Vocational High School Health Services, Giresun University, Giresun, Turkey

**Keywords:** *Hibiscus rosa-sinensis*, renewable energy, photovoltaic cell, black dye, open-circuit voltage

## Abstract

A new parabolic function for I-V curves’ analysis has been proposed. The new “analytical tool” provides a simple way to describe photophysical processes at an approximately monolayer surface of a dye-sensitized solar cell. It may now be possible to estimate factors such as hole–electron recombination, surface defects, and electron diffusion at the semiconductor layer. The theoretical approach that was previously reported by our group for predicting the photovoltaic performance of potential dye sensitizers has also been validated. The experimental photovoltaic and DFT/TD-DFT data of apigenin and those of the highly rated black dyes were used for the validation.

## Introduction

For more than 3 decades, scientists have been continuously and intensely engaged in the search for suitable dye systems to be used as sensitizers in the fabrication of highly efficient dye-sensitized solar cells (DSSCs) (Mehmood et al., 2014; Muchuweni et al., 2020). The target DSSCs are expected to produce overall efficiency of similar magnitudes as those of silicon-based solar cells (SBSCs) (Kwon et al., 2016; Gong et al., 2017). This, however, has not been achieved as most of the already-synthesized dye systems lacked the optical property require to produce an equivalent photoelectrical efficiency as those of the conventional SBSCs (Nazeeruddin et al., 2001; Mehmood et al., 2014; Kwon et al., 2016; Gong et al., 2017; Muchuweni et al., 2020). Of the already-synthesized plethora of dyes, the tricarboxy–terpyridyl ruthenium complex, commonly known as the black dye, is still the best photosensitizer (Nazeeruddin et al., 2001). The overall efficiency of the black dye in DSSC, nonetheless, is still far less than the conventional SBSCs.

Power generation has been a major challenge to global communities on account of the cost and negative impacts of activities that culminate into an eventual power production. Hundreds of thousands of research reports have been published since Thomas Edison’s first power plant was commissioned in 1882 (Luo et al., 2015). The results of these research efforts have led to the development of various power generation technologies available today (Breeze, 2010; Luo et al., 2015). It is interesting to note that most of these technologies have associated heavy financial and/or environmental cost, making us to realize that power generation is not cheap (Breeze, 2010). Sources based on fossil materials or nuclear substances are few examples that show how financially and environmentally costly power production could be. Hydro, wind, ocean wave, and solar sources are now being considered as safe, alternative sources. Although, for most of the global communities, the accessibility of these new sources is still very low due to the cost of installation.

The sun appears to be the easiest and the most accessible source of renewable energy amongst the identified sources. It is inexhaustible, quiet, and adjustable to enormous applications (Ludin et al., 2014; ByranvandMalekshahi, 2016). The amount of solar energy that gets into the Earth’s surface at any moment has been estimated to have convertible power of about 120,000 TW; approximately 8,000 times higher than the present rate of the global energy consumption per year (Yin et al., 2012). Consequently, photovoltaic (PV) technology has been viewed as an important means of attaining a healthy environment and a sustainable global economy. It has the potential to offer a solution for the dwindling fossil energy reserves, as well as the current issues of climate change.

Despite the potential advantages that PVs offer, the cost of conventional highly crystalline silicon-based PVs is limiting the solar energy usage. Therefore, the harvesting and conversion of solar energy into electricity at low cost using abundantly available raw materials remains a major research focus. Chemistry is therefore expected to make valuable contributions in this regard, by providing environmentally friendly solutions, one of which is the “organic PVs” (OPVs). OPVs employ organic dyes for light harvesting and sensitization to produce electrical power (O’Regan and Grätzel, 1991; Grätzel, 2001; Grätzel, 2003; Grätzel, 2005; Yella et al., 2011).

The development of new organic dye OPVs has been dominated by natural photosensitizers, mostly because of their low cost, abundant supply, and sustainability (Gao et al., 2000; Hao et al., 2006; Fernando and Senadeera, 2008). The technology can be scaled up without running into raw material supply problem, giving it an advantage over the currently used silicon-based PVs, which uses inorganic materials that require highly specialized skill to fabricate. One of the earliest deployed natural dyes is 8′-apo-β-caroten-8′-oic acid bound to TiO_2_ (Gao et al., 2000). OPVs based on these dyes are expected to produce an overall efficiency of similar magnitudes as those of the conventional SBSCs (Gao et al., 2000; Tryk et al., 2000; Hao et al., 2006; Fernando and Senadeera, 2008).

Notably, most of the dyes isolated or synthesized so far have not been quite successful in giving the required optical property that could produce equivalent photoelectrical efficiency as those of the known SBSCs. We have, therefore, in this article, tried to provide explanation on why the majority of dyes previously used have not given the desired photovoltaic response; knowing that the performance efficiency of OPVs depend majorly on the applied dye sensitizer. This effort has further helped validate our previously published theoretical model (Sanusi et al., 2019), which can act as a viable tool for predicting dyes’ photovoltaic efficiencies.

**FIGURE 1:**
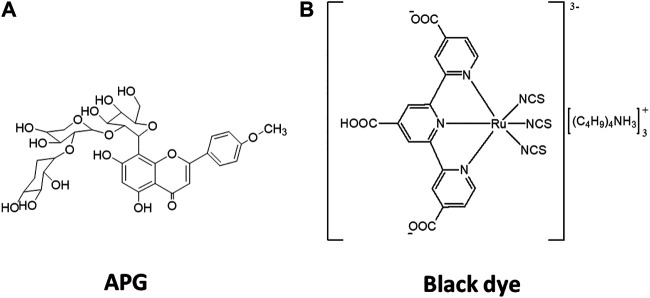
Isolated dye molecule with chemical name: Apigenin-4′-methylether-8C-β-D-glucosyl-(1'→6)-2″-O-β-[xylopyranosyl-[1′′′′→ 2‴)-O-β-xylopyranosyl **(A)**, and 2*D*-structure of the black dye **(B)**.

In this study, an apigenin (APG) derivative isolated from the leaf extracts of *Hibiscus rosa-sinensis* plant was employed as the source of our test natural dye (Figure 1A). *Hibiscus rosa-sinensis* plant is known to constitute considerable amount of dye pigments and had been used in organic solar cells’ fabrication previously (Fernando and Senadeera, 2008; Vankar and Shukla, 2011; Mansa et al., 2014). Comparisons between the computed and experimental photovoltaic properties of APG and the known tricarboxy–terpyridyl ruthenium complex (black dye = BD) have been made, as shown in Figure 1B (Nazeeruddin et al., 2001). To establish the relationship between theory and experiment, we used the computed and experimental photovoltaic data of APG and black dyes. While the experimental photovoltaic data of APG were obtained for this study, similar data for BD were taken from the literature (Nazeeruddin et al., 2001). The use of BD as the standard in this study was because it is currently the best synthetic photosensitizer for OPV cells (Wei et al., 2008; Hagfeldt et al., 2010; Feng et al., 2011; Akhtaruzzaman et al., 2013).

## Experimental Section

### Materials

#### Equipment and Methods

Electronic absorption spectrum of the isolated dye was collected on a Shimadzu UV-1800 spectrophotometer. ^1^H and ^13^C NMR data were collected on a Bruker Avance III 400 MHz spectrometer in DMSO-*d*_*6*_. Infrared (FT-IR) spectrum was recorded on a PerkinElmer Spectrum 100 FT-IR spectrometer. The cells’ I-V characteristics were determined using an intensity of ∼35.7 W cm^−2^, from a Newport 66245 Oriel lamp, which was coupled with a Kelthley 2400 multimeter. All solvents were distilled before use. TiO_2_ paste was prepared with a mixture of 95% ethanol and 15% acetic acid at a volume ratio of 1:1. The iodide/triiodide redox solution employed was prepared by weighing 10 g of KI in 100 ml of distilled water, after which 15% acetic acid solution of iodine (obtained with pure iodine crystals) was slowly added. The resulting solution was filtered and the filtrate was kept in a tightly stoppered amber bottle. Five cell samples (1–5) were fabricated with the APG dye at different concentrations of the dye. The dye solutions were prepared in 1:1 volume of 95% ethanol and 15% acetic acid.

Structures of APG and BD dyes are depicted in Figures 1A,B, respectively. Both the ground and excited state calculations on the structures were performed using the Gaussian 09 package (Frisch et al., 2010). Ground state optimization and vibrational frequency of APG were carried out using B3LYP hybrid functional with the 6-311 g(d) basis set. Mixed basis sets with effective core potential LANL2DZ and 6-311 g(d) for C, H, N, O, and S atoms were used for BD at the B3LYP level. TD-DFT vertical excitation energies and the oscillator strengths were computed for the two dyes at the same level of theory employed for their optimization and frequency calculations. Solvent effects were incorporated in both the DFT and TD-DFT calculations using the integral equation formalism polarizable continuum model (IEF-PCM) (Tomasi et al., 2005) with acetonitrile (AcCN) and ethanol (EtOH) as solvents. Theoretical photovoltaic and photophysicochemical parameters of the two dyes were estimated as described in our previous article (Sanusi et al., 2019). The experimentally determined TiO_2_ conduction band (CB) edge reported by Xu and Schoonen (2000) was employed in the determination of the LUMO-TiO_2_ CB (*δ*_p_) gap as described previously (Sanusi et al., 2019).

## Results and Discussion

### Structural Characterization of the Isolated Dye Molecule

The APG molecule (412 mg) was isolated as a yellow amorphous powder. The ^1^H NMR and ^13^C NMR spectra exhibited signals due to aromatic systems and sugar moieties. The ^1^H NMR spectrum of APG showed a pair of ortho-related protons, indicating the AA'BB' aromatic ring system due to ring B of flavonoid. The ^1^H NMR spectrum of APG suggests one flavone unit with signals corresponding to many sugar moieties and one methoxy (-OCH_3_) group. It also exhibited one down field peak at δ_H_ 13.33 ppm for chelated-OH, a pair of doublets (J = 8.8 Hz each) indicating *para* substituted ring B, and a singlet at δ 6.79 ppm due to H-3 on ring C. We observed another singlet at δ 6.48 ppm, which was assigned to H-6. A singlet observed at δ_H_ 3.87 ppm confirmed the presence of a methoxy (−OCH_3_) group and was assigned to position 4′ on ring B of the flavone unit. The anomeric proton of the first sugar unit appeared as a broad signal at δ_H_ 4.87 ppm (J = 7.2 Hz, indicating *β*-configuration), while the remaining sugar protons appeared between δ 3.13 and 4.20 ppm. The ^13^C NMR spectrum of this dye revealed two separate singlets corresponding to C-3 and C-6 positions of flavone nucleus, indicating a substituted C-8. The absence of meta-related coupling suggested a C-8 substituted flavone. Correlation between the anomeric proton δ_H_ 4.92 of the first glucose sugar unit and C-6 (δ_C_ 104.9) of the aglycone confirmed the attachment of the anomeric sugar carbon to the C-6 position of the flavone nucleus. Based on the 1D-NMR data and comparison of the data given in the literature (Nawwara et al., 2014), it could be concluded that the structure of APG is a 4′-methoxy derivative of vitexin (apigenin-4′-methylether-8C-glucopyranoside). The proton and carbon-13 NMR results have been summarized in Table 1.

**TABLE 1:** ^1^H (400 MHz, DMSO- *d*_*6*_) and ^13^C (100 MHz, DMSO- *d*_*6*_) NMR data of isolated dye molecule (APG) and vitexin from the literature.

APG			^a^Vitexin 2ʹʹ-O-β-[xylosyl-(1ʹʹʹʹ→2ʹʹʹ)-O-β-xylopyranosyl] from the literature	
Position	1H (ppm) *J* (Hz)	^13^C (ppm)	1H (ppm) *J* (Hz)	^13^C (ppm)
2		164.3		163.72
3	6.79 s	102.7	6.76, s	102.44
4		182.3		182.00
5	13.33 s, (5-OH)	161.3		161.20
6	6.48 s	98.1	6.25, s	98.30
7		163.5		161.69
8		104.9		103.80
9		155.3		156.50
10		104.7		104.53
1ʹ		121.7		121.50
2ʹ	8.03, d, (8.8)	129.1	7.95, d, (8.0)	128.78
3ʹ	6.91, d, (8.4)	115.9	6.98, d, (8.0)	115.98
4ʹ		161.5		
5ʹ	6.91, d, (8.4)	115.9	6.98, d, (8.0)	115.98
6ʹ	8.03, d, (8.8)	129.1	7.95, d, (8.0)	128.78
4-(OCH3)	3.87 s	56.7		
Glucosyl signals				
1ʹʹ	4.72, d, (8.4)	71.4	4.62, d, (8.5)	71.22
2ʹʹ		82.9		83.26
3ʹʹ		78.4		79.30
4ʹʹ		69.9		69.80
5ʹʹ		81.9		82.44
6 ʹʹ		61.3		61.95
Xylosyl signals				
1ʹʹʹ	4.87, d, (7.2)	96.4	4.80, d, (7.0)	97.73
2ʹʹʹ		81.8		83.51
3ʹʹʹ		73.1		73.25
4ʹʹʹ		67.8		68.13
5ʹʹʹ		64.4		65.45
Xylosyl signals				
1ʹʹʹʹ	4.92, d, (9.2)	104.2	4.87, d, (7.0)	103.86
2 ʹʹʹʹ		74.4		73.80
3 ʹʹʹʹ		76.8		77.31
4 ʹʹʹʹ		69.2		68.12
5 ʹʹʹʹ		69.3		68.46

a Data for vitexin derivative were obtained from reference (Nawwara et al., 2014).

**FIGURE 2:**
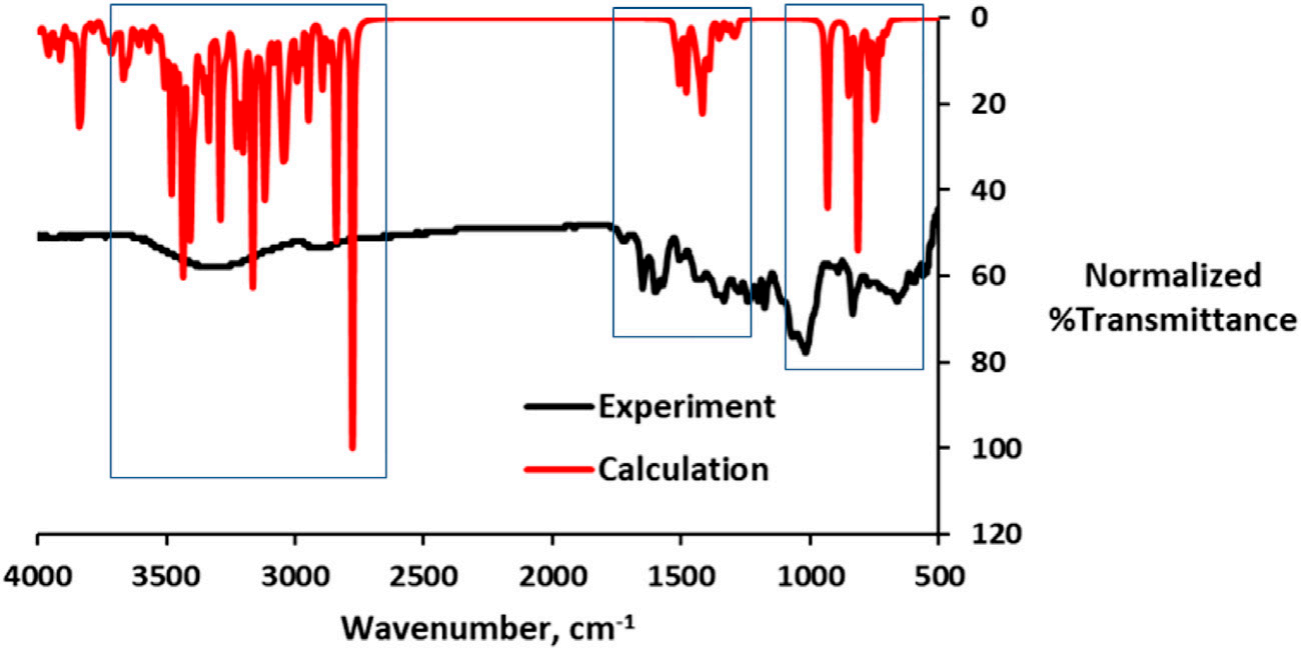
Overlay of the calculated and experimental IR data (red = computed and black = experiment).

It has been reported that specific functional groups are required for dyes to be effectively adsorbed onto the TiO_2_ thin film (Chang et al., 2013). A previous study by Ahmad and Nafarizal (2010) also reported that some functional groups such as hydroxyl groups (–OH) and carbonyl groups (–CO) are important in providing points of attachment to the TiO_2_ surface. Table 2 shows the FT-IR spectra with diagnostic absorption bands within the wave band of 4,000–400 cm^−1^. The FT-IR of the APG isolated from the *Hibiscus rosa-sinensis* leaf extract revealed the presence of CH_3_ and CH_2_ vibrations at 2,930 and 2,634 cm^−1^, respectively. Moreover, vibrations of C=O at 1716 cm^−1^, C–O at 1,043 cm^−1^, C=C at 1,651 cm^−1^, and O–H at 3,311 cm^−1^ were also observed. Calculated IR data for the APG is presented in Figure 2 alongside the measured data. The computed IR data are in good agreement with the experiment, suggesting that the theoretical method adopted is suitable in the description of the molecular structure.

**TABLE 2:** IR absorption band of APG isolated from *Hibiscus rosa-sinensis*.

Functional group	APG absorption bands (cm^−1^)
O-H	3,311
C=C	1,651
(sp^3^) C-H	2,893, 2,634
C=O	1716
C-O	1,043

### New Parabolic Function for Analyzing I-V Curves

Initially, it was thought that the experimental photovoltaic parameters (J_sc_ and V_oc_) and the overall efficiencies (*%PCE*_expt_ and *%η*_global_) of the fabricated APG cells would increase with increasing dye concentration. Interestingly, what was observed was different from this expectation (Table 3). There was no clear trend in the observed photovoltaic data with respect to concentration. To explain this observation, a parabolic model, from which could be obtained certain physical parameters that may be useful in understanding the underlining photoelectrical processes, was proposed (Eq. 1).

$$J_{\text{eff}}=\gamma +\beta V-\delta V^{\alpha },$$ (1)

$$V_{\text{eff}}=\beta V-\delta V^{\alpha }.$$ (2)

**TABLE 3:** Experimental photovoltaic properties of the APG-based DSSCs at different concentrations as compared to the properties of the reported BD-based DSSC.

Cells	APG in EtOH at various conc. [mol. dm^−3^]	J_sc_ [µA/cm^2^]	V_oc_ [V]	*%PCE*_expt_	*%η*_global_	*γ*	*α*	*β*	*δ*	J_max,eff_
1	1.1 × 10^−2^	0.125	0.899	3.96 × 10^−7^	1.49	0.125	5.50	4.53 × 10^−2^	0.285	0.007
2	2.1 × 10^−2^	0.141	1.000	1.65 × 10^−7^	0.84	0.141	9.33	1.83 × 10^−5^	0.088	0.053
3	3.2 × 10^−2^	0.141	0.820	4.58 × 10^−7^	1.53	0.141	9.54	1.96 × 10^−5^	0.720	0.033
4	4.2 × 10^−2^	0.129	0.842	7.46 × 10^−7^	1.98	0.129	10.02	0	0.379	0.061
5	6.2 × 10^−2^	0.128	0.880	3.09 × 10^−7^	1.46	0.128	29.68	1.84 × 10^−5^	3.744	0.045
^a^BD	∼2.0 × 10^−4^	20.5 × 10^3^	0.720	—	10.4	—	—	—	—	—

a Data for BD was obtained from reference (Nazeeruddin et al., 2001). *J*_max,eff_ is the maximum effective charge factor that influence the power output of the DSSC assuming that the intrinsic potential has a magnitude equivalent to the V_oc_. **1–5** represents the cells fabricated using different dye concentrations.

**FIGURE 3:**
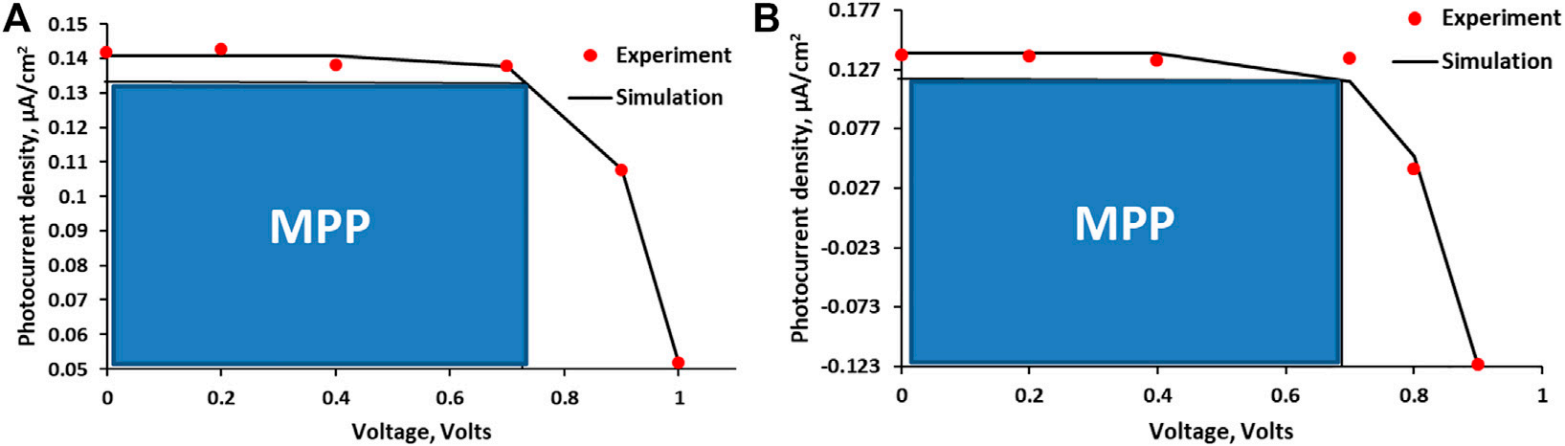
I-V profiles (experimental and simulated) for cell 2 **(A)** and cell 3 **(B)** as representatives (MPP, maximum power point). The solid line represents the fitting curve obtained with Eq. 1.

The representative experimental I-V profiles for the APG-based cells are presented in Figure 3, with the solid line representing the fitting curve. The graphs were fitted to the parabolic function described in Eq. 1. The model assumes that the effective potential (V_eff_), Eq. 2, is a function of some intrinsic potential V, and some variable factors, α, β, and δ. The intrinsic potential V has been assumed to depend on dye electron injection efficiency (*φ*_inj_), velocity of charge transport on the semiconductor medium (*ω*), and the amount of dye that is available (*n*) for sensitization. Both $J_{\text{eff}}$ and $V_{\text{eff}}$ in this case are unit-less values that describe charge and force factors, respectively. The variables, α, β, γ, and δ, which were obtained by fitting Eq. 1 to the experimental I-V curves (Figure 3), may be described as the diffusion, hole–electron recombination, charge transfer, and surface defect factors, respectively, with all of them influencing the overall efficiencies of the DSSCs (%PCE and %*η*_global_). γ was found to be 1 × 10^6^ fold higher in magnitude than the experimental short circuit current (J_sc_) for each of the cells 1–5. The diffusion factor α was observed to increase proportionally with the dye concentration (Table 3). The maximum effective charge factor (J_max,eff_) was estimated by assuming that the intrinsic potential has a magnitude equivalent to that of V_oc_, that is, the experimental V_oc_ was taken as V in Eq. 1. J_max,eff_ values were found to vary with α, β, γ, and δ. The cell’s overall efficiencies were found to depend on the J_max,eff_ values (Table 3). As indicated in Table 3, cell 4 composing of 4.2 × 10^−2^ mol dm^−3^ of APG produced the highest J_max,eff_ value and global efficiency of 0.061 and 1.98%, respectively. A slight decrease in the value of β was also found to result in a significant increase in the J_max,eff_ values (Table 3).

The fit factors α, β, γ, and δ, especially, were shown to affect the power conversion (%PCE), and the global (%*η*_global_) efficiency values of the cells which have generally followed a noticeable trend for which the values in cell 2 < cell 5 < cell 1 < cell 3 < cell 4. It is worthy of note that cell 5 which contained the highest amount of dye gave a δ value of 3.744. This value is the highest of the five cells (Table 3), suggesting that it has the highest degree of surface defects, which could explain why it has one of the lowest power efficiencies. This high degree of surface defects might have resulted from the aggregation of dye molecules due to increasing dye concentration. With these fitting parameters, it may now be possible to explain I-V curves of different DSSCs.

### Validation of Previous Theoretical Model

**FIGURE 4:**
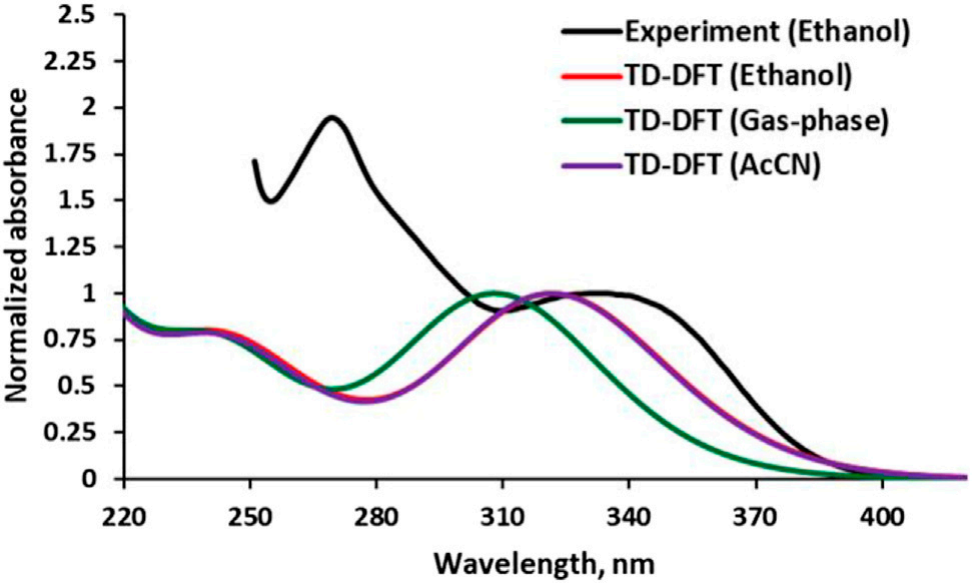
Experimental (conc. ∼1.10 × 10^−5^ mol/dm^3^) and calculated electronic absorption spectra of APG in different media.

The observed electronic absorption property of the APG in Ethanol (EtOH) is depicted in Figure 4 along with those obtained by TD-DFT methods in the gas phase, EtOH, and acetonitrile (AcCN). The observed spectrum showed a broad absorption band covering between 320 and 350 nm and peaked at ∼340 nm (λ_max_). The computed solution-phase electronic spectra showed similar pattern with the experiment but with slight blue-shifting compared to the latter. The agreement between the solution-phase electronic properties obtained *via* computation and the one by experiment indicates that the computational method employed is suitable. Solvent effects are noticeable in the computed λ_max_ when comparing APG in gas and solution phases (Figure 4). The superimposed computed spectra of APG in EtOH and AcCN are red-shifted relative to the gas phase.

It is understood that the lack of sufficient chromophoric groups in the isolated APG would put it at disadvantage for the proposed application, which requires high extinction coefficient over a wide spectral range, covering the visible to the near infrared region. Nonetheless, APG was an ideal choice of sample to validate the previous theoretical model for the prediction of dye’s photovoltaic properties prior to the use of the dye (Sanusi et al., 2019). On the other hand, the BD which has shown relatively higher extinction coefficients (Nazeeruddin et al., 2001), spanning from the visible to the infrared region, is expected to show superior photovoltaic responses compared to APG, and so could serve as a suitable positive reference to validate the theoretical method (Sanusi et al., 2019). The experimental photovoltaic data showed that BD is in manifold better as a photosensitizer than the APG, with the overall efficiency (*%η*_global_) value being 5.25 times higher than that of the best APG-based cell (cell 4) (Table 3).

**FIGURE 5:**
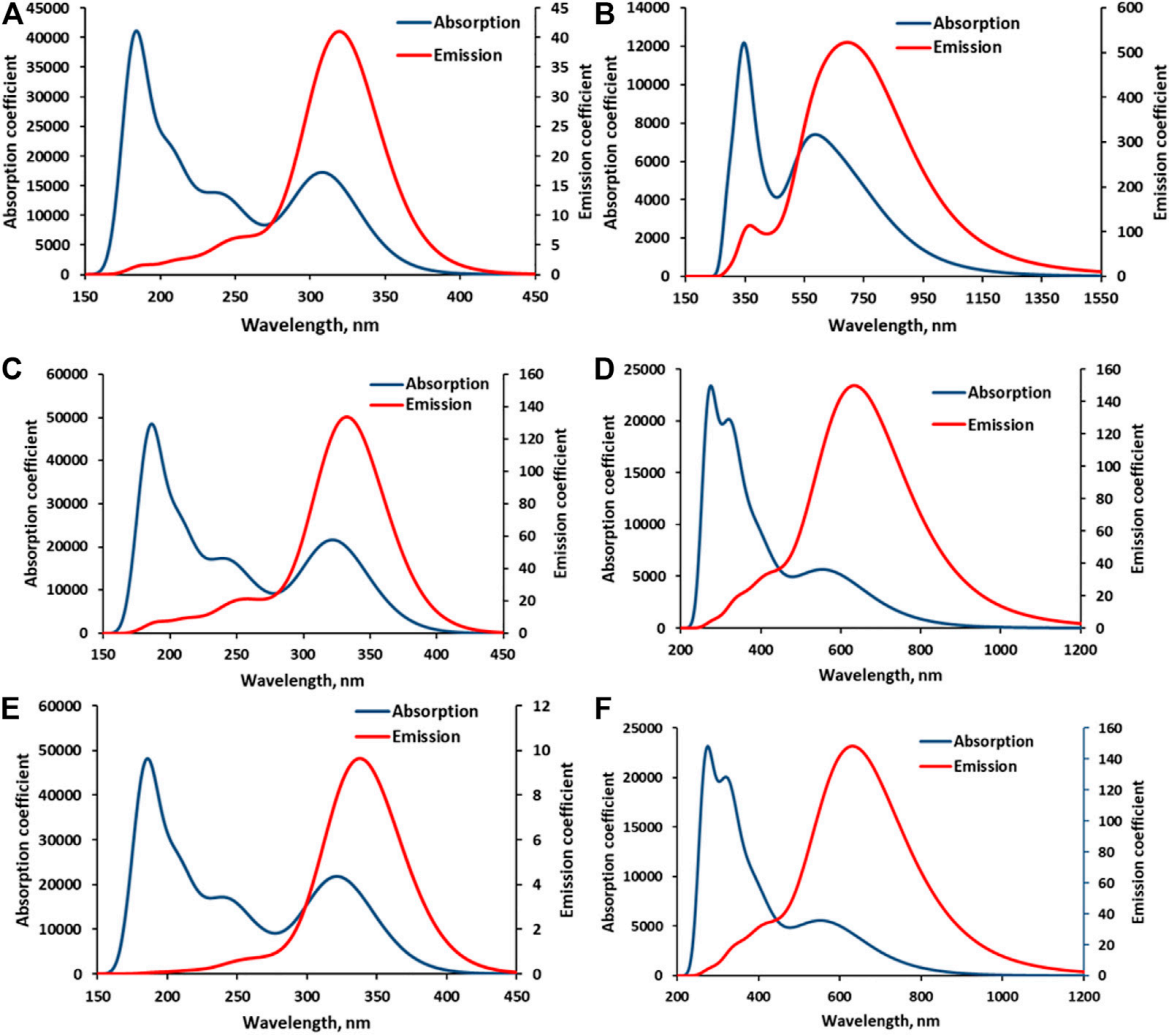
Calculated electronic absorption and emission spectra of APG and BD in various media. **(A)** = APG in the gas phase, **(B)** = BD in the gas phase, **(C)** = APG in EtOH, **(D)** = BD in EtOH, **(E)** = APG in AcCN, and **(F)** = BD in AcCN.

The computed absorption–emission spectral curves presented in Figure 5 show the longer spectrum range covered by both absorption and emission curves of BD when compared to APG in the three media—gas, EtOH, and AcCN. The calculated photovoltaic parameters, which include light-harvesting (LHE), electron injection efficiency (*φ*_inj_), and incident photon conversion efficiency (IPCE) are mostly higher in BD than APG in the three media, except in the gas phase where the charge collection efficiency (*η*_c_) is higher for APG. The *δ*_p_ term (potential gap) which is excessively high for BD in the gas phase could be responsible for the lower *η*_c_. It may thus imply that the excited state BD is highly unstable in the gas phase, but much more stable in solution (Table 4). Overall, the conclusions from the computed data (Table 4) that BD is a better sensitizer for DSSCs do compare favorably with those of experiments (Table 3). This study, by comparing the experimental I-V data of APG and BD, to those predicted by our previously reported theoretical method, has confirmed the validity of the theoretical method. The method predicted that BD is a better sensitizer compared to APG, just as the experiments have done for the considered media phases, except in the gas phase. It is, however, worthy of note that there is no experimental gas phase photovoltaic data for these two sensitizers yet; hence, it would be impossible to know the validity of the theoretical result that shows APG as a better sensitizer in terms of *η*_c_ in the gas phase. The free-energy of injection (∆G_inj_) is generally negative for both dyes in the three media, suggesting that their electron injection from the LUMO to the TiO_2_ CB edge would be spontaneous (Table 4).

**TABLE 4:** Computed photophysicochemical and photovoltaic parameters of the APG and BD in gas and solution phases.

Sample	*f* × 10^−3^	LHE	∆G_inj_	*φ*_inj_ × 10^−4^	δ_p_ (eV)	*IPCE*_calc_ × 10^−15^	*η*_c_ × 10^−12^
APG in the gas phase	8.90	0.0203	−1.445	9.86	2.23	3.16	158
BD in the gas phase	93.7	0.194	−7.375	628	7.69	159	13.1
APG in EtOH	88.7	0.185	−1.574	26.7	2.12	1.76	3.57
BD in EtOH	135	0.267	−1.187	111	1.69	16.4	5.52
APG in AcCN	87.1	0.182	−1.577	34.6	2.11	6.46	10.3
BD in AcCN	137	0.271	−1.112	111	1.62	51.8	17.3

## Conclusion

A natural dye, an APG derivative, has been isolated from the leaf extracts of *Hibiscus rosa-sinensis* plant. The full structure of the isolated dye molecule has been elucidated by ^1^H and ^13^C NMR, FT-IR, and UV spectroscopic techniques. The I-V characteristics of the dye have also been measured, with further interpretation of the data given by the use of a new parabolic function. Activities at an approximately monolayer semiconductor surface, such as hole–electron recombination, surface defects, charge transfer, and electron diffusion factors could be determined from this model. The theoretical approach previously reported by our group for the prediction of dyes’ photovoltaic performance has been validated. The experimental photovoltaic data obtained for the APG- and BD-based cells, together with the TD-DFT data of these dyes, were employed in the method validation.

## Data Availability

The original contributions presented in the study are included in the article/Supplementary Material; further inquiries can be directed to the corresponding author.
